# Quantitative response of healthy muscle following the induction of capsaicin: an exploratory randomized controlled trial

**DOI:** 10.1186/s13063-020-04937-4

**Published:** 2020-12-11

**Authors:** Valerie Evans, Michael Behr, Kei Masani, Dinesh Kumbhare

**Affiliations:** 1 Institute of Biomaterials and Biomedical Engineering (IBBME), University of Toronto, Toronto, Ontario Canada; 2 Toronto Rehabilitation Institute, University Health Network, Toronto, Ontario Canada; 3 Department of Medicine, Division of Physical Medicine and Rehabilitation, Toronto, Ontario Canada

**Keywords:** Myofascial pain syndrome, Ultrasound, Image texture, Electromyography, Central sensitization, Trigger points

## Abstract

**Background:**

Myofascial pain syndrome (MPS) is a prevalent chronic pain disorder primarily characterized by myofascial trigger points (MTrPs). There is limited knowledge on the pathophysiology and mechanisms underlying MTrP and its development. Research has previously demonstrated the identification of MTrPs using ultrasound and vibration sonoelastography, although there is some contradictory evidence regarding if MTrPs present as hyper or hypoechoic regions. Electromyography (EMG) investigations of MTrP have demonstrated that MTrPs are usually located proximal to innervation zones where the peak surface EMG signals are obtained from. Central sensitization has been proposed as the primary mechanism underlying MTrP development. Central sensitization is associated with hyperexcitability of neuronal responses to normal or noxious stimuli. There is a need for a study that measures ultrasound image textural changes and motor unit activity responses in the muscle following sensitization. The purpose of this study is to determine whether sensitizing healthy muscle using capsaicin induces a regional change in image texture variables within the specific and surrounding muscles, as well as the motor unit frequency and amplitude changes that accompany them. This is an exploratory trial that aims to provide preliminary evidence on whether central sensitization is a direct cause of taut band and MTrP development.

**Methods:**

Ethical approval was obtained from the University Health Network (UHN) Research Ethics Board. This proposed study is a single centered, factorial, randomized placebo-controlled trial with two independent variables, depth of capsaicin application and dose of capsaicin, for a total of six treatment arms and three control treatment groups.

**Discussion:**

This will be the first study that assesses the B-mode ultrasound image texture of induced sensitized muscles and will provide more evidence on muscle motor unit activity and regional changes of central sensitization. Findings from this study may support one of few hypotheses proposed delineating the involvement of central sensitization in the development of trigger points.

**Trial registration:**

National Institutes of Health ClinicalTrials.gov NCT03944889. Registered on May 07, 2019

## Background

Myofascial pain syndrome (MPS) is a prevalent chronic pain disorder primarily characterized by myofascial trigger points (MTrP). MTrPs are stiff taut bands of muscle distinguished by hypersensitivity on palpation and local twitch responses on snapping palpation [1]. A current clinical challenge in chronic pain care, applicable to myofascial pain, is early detection and effective treatment targeting the underlying pathophysiology.

There is limited knowledge on the pathophysiology and mechanisms underlying MTrP and its development. Ultrasound studies have been able to characterize trigger points [2, 3]; however, there are some contradictory findings on whether these areas are hyperechoic or hypoechoic [3, 4]. More recently, texture feature analyses have been shown to differentiate between healthy and MPS as well as active and latent trigger points [2]. Trigger points are typically located within the region of the muscle belly, where there is a high distribution of motor endplates [5, 6]. Seventy-one percent of acupoint locations in the body, which are regions associated with motor points, motor endplates, or major motor nerve pathways, overlap with common MTrP [7].

Electromyography (EMG) investigations of MTrP have demonstrated that MTrPs are usually located proximal to innervation zones where the peak surface EMG signals are obtained from [6]. Motor endplates at MTrP present with spontaneous endplate activity (SEA) associated with excessive acetylcholine release [8, 9]. In latent and active trigger points, SEA is characterized by continuous low amplitude action potentials. Active MTrPs, which are more painful clinically, elicit intermittent spikes in addition to SEA. This continuous activity is thought to overexert and hyper contract the muscle resulting in the presentation of a taut band [10]. Studies assessing the contractility of MTrP have found increased muscle fatigability in active and latent MTrP relative to unaffected muscles. Furthermore, it has also been demonstrated that there is increased fatigability and EMG activity in active MTrP relative to latent ones [11]. This data suggests that perturbations in the efferent system involved in muscle control are a vital component of the MTrP pathophysiology.

Central sensitization has been proposed as the primary mechanism underlying MTrP development. Central sensitization is associated with hyperexcitability of neuronal responses to normal or noxious stimuli [12]. Furthermore, this concept offers an explanation of the afferent, efferent, and abnormal signal modulation at the spinal cord level [12, 13]. Patients with myofascial pain present with hyperalgesia and decreased pain threshold, i.e., signs of central sensitization, particularly at the region of MTrP [8, 14]. Experimental evidence has demonstrated that afferent nociceptive fibers at the region of MTrP are sensitized. Neurons in the dorsal horn segment corresponding to the location of MTrP are also sensitized, manifesting clinically as hyperalgesia within the dermatome affected by MTrP [15]. This is evidenced by Kim et al.’s [15] findings whereby they reported transcutaneous electric stimulation increased MTrP pressure pain thresholds when applied at a remote region within the same dermatome. Srbely [1] further presented evidence of a sensitization arc that maintains the MTrP contracture. They experimentally induced sensitization at a remote region in the dermatome wherein a latent MTrP is located using topical capsaicin, and subsequently found a decrease in pressure pain thresholds at the MTrP.

Investigations assessing neuronal responsivity to noxious stimuli have demonstrated increased sensitization at the region of primary nociception and the development of a secondary nociceptive field [13–15]. This has been confirmed using EMG and quantitative sensory testing methods to assess the activity of sensory nociceptive, mechanoheat, and chemoreceptive nerve fibers following the injection or application of capsaicin [16–18]. Many other factors are likely involved in the chronic pain patient including disordered ascending and descending signals within the central nervous system, termed nocioplastic pain by the International Association for the Study of Pain (IASP) [19–21]. Dideriksen et al. [22] and Falla et al. [23] demonstrated that motor unit potentials reduce in amplitude following the injection of nociceptive hypertonic saline into the upper trapezius muscle. This is in line with the EMG characteristics of MTrPs cited earlier [10, 11]. However, other studies, such as the study directed by Birch et al. [24], present contradictory results, indicating that no significant differences in motor unit discharge rates were found. Studies assessing the EMG activity of trigger points failed to locate the aberrant activity that characterizes MTrP using surface EMG but were able to demonstrate the presence of SEA, intermittent spikes in active MTrP, as well as activity associated with local twitch responses using intramuscular EMG recordings due to the method’s specificity [8].

In regard to MTrP structure, Shankar et al. were able to identify taut bands of MTrPs using 3D and 2D ultrasound [25]. They discovered that the muscle fascicles surrounding the taut band presented a different appearance than the adjacent healthy muscles. The study identified MTrPs as hyperechoic regions, as in another study confirmed by Lewis et al. [4]. In contrast to these studies, Sikdar et al. noted that MTrPs appear as hypoechoic elliptical regions within the ultrasound image [26]. Although there is some evidence on the changing structure of myofascial trigger points, to the best of our knowledge, there has been no evidence of how central sensitization affects muscle structure through image analysis. There is a need for a study that measures specific B-mode ultrasound textural changes within sensitized areas, as well as motor unit activity responses in the muscle following sensitization.

The purpose of this study is to determine whether sensitizing healthy muscle using capsaicin, a chili pepper extract, induces a regional change in ultrasound texture features of the targeted muscle, or of the muscles in that are in close proximity. This regional change will be accompanied by EMG recordings to confirm the presence of abnormality. Central sensitization will be induced using topical capsaicin and injectable capsaicin at two different concentrations and tested against placebo. This is an exploratory trial that aims to provide preliminary evidence on whether central sensitization is a direct cause of taut band and MTrP development.

Our specific research questions are the following:

Principle research question and hypothesis are as follows:

Does capsaicin induced sensitization (applied topically, intramuscular and intrafascial injections) elicit a change in motor unit action potential (MUAP) amplitude by approximately 20 to 25% within the region of experimentally induced central sensitization?

Our hypothesis for this portion of the study is that sensitization modifies the anterior horn cell activity which will be measured by the amplitude of the potentials. We hypothesize a dose response effect to be elicited with experimentally induced sensitization.

Secondary research questions and hypotheses are as follows:

- Does sensitization (induced by capsaicin topically, intramuscular and intrafascial injections) cause a significant regional change in the texture features of the targeted muscle or surrounding muscles? Our hypothesis is that that there will be a structural change within the sensitized or adjacent muscles.
- Does capsaicin-induced sensitization (applied topically, intramuscular and intrafascial injections) elicit continuous electrical activity as observed in MTrPs? This tests the hypothesis that sensitization creates the presence of continuous low amplitude action potentials.
- Does capsaicin-induced sensitization (applied topically, intramuscular and intrafascial injections) influence the rate of recruitment of motor units in the muscle within the region induced? The recruitment of motor units is normally expected to follow the Henneman size principle [27–30]. Our hypothesis for this portion of the study is that sensitization will cause an aberration in recruitment. If this is demonstrated, then there is modification of the normal processing at both dorsal and ventral horns of the spinal cord.
- Is there a location-dependent EMG response to capsaicin-induced sensitization? In other words, do the EMG responses from muscles that lie anatomically distant from the location of the site of capsaicin application (topical, intramuscular injections, and intrafascial injection) have changes from their baseline calculations? This would test which type of afferents has an influence on the anterior horn cells that lie distant to the ones that are supplying muscle fibers in the vicinity of the stimulated sensory afferents.
- Is there a location-dependent ultrasound texture feature response to capsaicin-induced sensitization? In other words, do the ultrasound texture feature responses from muscles that lie anatomically distant from the location of the site of capsaicin application (topical, intramuscular injection, and intrafascial injection) have changes from their baseline calculations? This would test which type of afferents has an influence on the anterior horn cells that lie distant to the ones that are supplying muscle fibers in the vicinity of the stimulated sensory afferents.
- Is there a dose-dependent EMG response of motor units to capsaicin-induced sensitization (applied topically and injected via intramuscularly and intrafascial)? Here, the hypothesis of causality will be assessed in a preliminary manner. If there is a relationship, then further experiments that more thoroughly assess causality will be needed.

## Methods and design

### Study design

Ethical approval is approved by the University Health Network (UHN) Research Ethics Board.

This study is registered under the National Institutes of Health ClinicalTrials.gov.

This proposed study is a single centered, factorial, randomized placebo-controlled trial with two independent variables, depth of capsaicin application and dose of capsaicin, for a total of nine treatment arms. The first between groups variable will be topical capsaicin application and injectable capsaicin—including an intramuscular injection as well as intrafascial injection. Participants will be split between these three groups. Within each partition, there will be three treatments: control, 50 μg, and 100 μg. One hundred micrograms reported to be an effective high dose of capsaicin [16]. The control group will receive a topical skin lotion which is inert and has no sensitization effect, or an inert injection (for the intramuscular or intrafascial groups). An equal number of participants will be allocated to each of the nine treatment groups using an electronic randomization generator. Block randomization will be used to ensure equal allotment into each group. An alternate member of the research team will conduct the randomization schedule a priori. Participant allocation will be concealed by placing their assignment in an envelope and delivered to participants by the same member of the research team uninvolved in the measurement or randomization protocols. Participants and investigators will be blinded to the delivered dose; however, the type of capsaicin delivery cannot be blinded from either participants or investigators. The member of the research team conducting the randomization schedule and concealing allocation will have knowledge of and keep track of the doses contained in the containers and vials of the topical and injectable capsaicin, respectively.

They will deliver the appropriate dose to the team member implementing the experimental protocol to ensure there is blinding with respect to dose. This individual will not be involved in participant recruitment.

### Participants

#### Recruitment

Participants will be recruited from UHN and the University of Toronto clinics, employees, volunteers, visitors, students, and external participants. UHN is a tertiary healthcare center with eight hospitals located in Toronto, Canada, and the University of Toronto is an academic institution with a main campus located in close proximity to the main UHN hospitals in Toronto. Advertisements, flyers, and in-person recruitment will be employed to collect the necessary sample size for the study. The recruitment procedures will run in line with the approved guidelines from the UHN research ethics boards. Participants will not be coerced into participating and will be informed that they have the right to withdraw at any time during the experimental procedures. They will also be informed they have the right to withdraw their data prior to publication. Participants will be briefed and consented on recruitment prior to commencing the study procedures. Participant treatment allocation will be recorded on a separate document until all of the data collection is complete and the data is analyzed.

#### Eligibility criteria

Female or male participants who meet the following inclusion criteria will be included in the study: (1) aged, 20–60; (2) participants are healthy with no past musculoskeletal and neurological medical history; (3) a visual analog score as a measure of current pain below 3 indicating low pain severity, however, ideally who experiences no pain; (4) body mass index > 19 < 25; (5) have sufficient command of the English language to provide informed consent and to understand the study protocols; and (6) participants agree to sign a consent to volunteer for the research.

#### Exclusion criteria

Participants who meet one or more of the following criteria will be excluded: (1) absence of pain,(i.e., headaches/migraine, toothaches, pain from sports injuries) at the time of recruitment; (2) physical examination detection of myofascial trigger points; (3) participants present with a history of pain-related disturbances such as poor sleep, cognitive disturbances, and psychiatric disorders; (4) history of general medical disorder that may affect the outcome of the study such as diabetes mellitus; (5) and a history of cervical radiculopathies or (6) history of inflammatory arthropathy.

### Experimental protocol

This experimental protocol will be carried out at the Kumbhare Lab, Toronto Rehabilitation Institute, to ensure any medical emergencies can be promptly cared for. Following the completion of the preliminary intake forms and answering any potential participant’s question(s), we will explain the study protocol and then, informed consent will be obtained. Each participant will be seated upright with their hands comfortably on their lap in a chair that has a high supportive back. They will be asked to relax their neck and shoulder muscles. A member of the research team will then apply the inclusion and exclusion criteria. The presence of a MTrP will be assessed by palpation of the upper trapezius muscle since this is the current method utilized in clinical practice. If there is a MTrP, then they will be asked to withdraw from the study since they do not satisfy the inclusion and exclusion criteria.

Participants will sit on a chair with one arm extended downward, and with the other arm on an armrest of the chair. The extended arm will be fixed by a wristcuff and a chain down to the floor, with a loadcell between the floor and the end of the chain. The loadcell will measure the exerted force. The anatomical location for ultrasound probe and electrode placement will be identified for each participant. The ultrasound probe will be placed on 3 muscles: the trapezius, the supraspinatus, and the infraspinatus. Two ultrasonic pictures will be taken of each. Care will be taken to image the region of the muscle that was not mechanically impacted by the insertion of the needle or injection of the substance. The area will then be cleaned with alcohol and water.

**Fig. 1.**
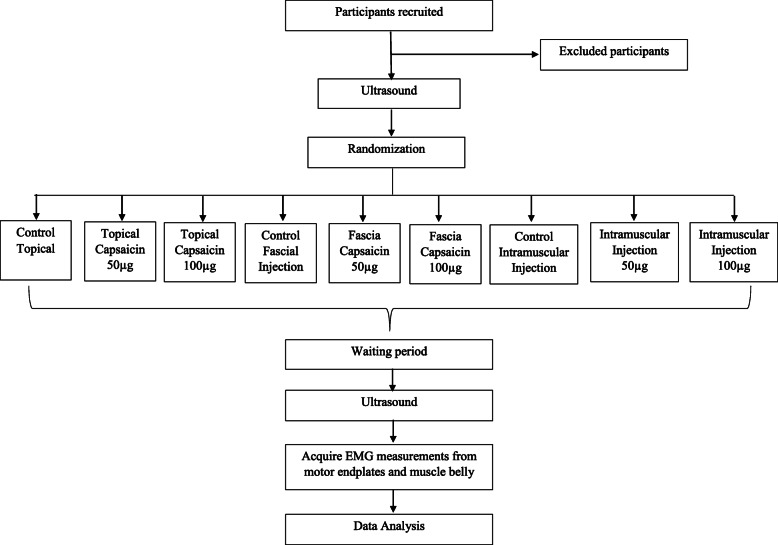
Timeline of study activities

The Delsys Galelio surface sensor and the intramuscular needle electrode will be placed directly on the trapezius’ identified area. Participants will be asked to gently contract their trapezius muscle. They will be instructed to perform a gradually increasing contraction in isometric condition, in a controlled manner with a monitor showing the exerted force as well as the target force. They will hold this contraction at 30% of their maximal voluntary contraction and then perform a gradually decreasing contraction to rest. This will be performed four times for each participant before as well as after the intervention. The placement of the injection needles will be verified by ultrasound guidance. The intramuscular needle will then be removed and participants will be bandaged and cared for appropriately by the expert physician performing the experiment. Following this, ultrasound pictures will be taken again from the same muscles mentioned previously. Participants will be re-examined to determine if there were any adverse effects from the experimental procedures. If any occurred, then these will be carefully managed by the medical members of the research team and recorded. Participants will be asked to remain at the lab for an additional 30 min to ensure they are well prior to leaving (Fig. 1).

### Central sensitization and measurement techniques

#### Inducing central sensitization

Central sensitization will be induced using capsaicin according to the established technique [16, 31, 32]. It induces increased pain severity at high doses relative to heat and elicits dose-dependent pain responses, allowing for more easily quantifiable measurements [33].

Capsaicin delivery induces both primary and secondary nociception suggesting that central mechanisms are involved in the nociceptive response, also termed as central sensitization [16, 17].

Previous evidence has confirmed that capsaicin can sensitize nociceptive and mechanoheat receptors outside of the region of primary hyperalgesia, with speculation that it can also sensitize chemonociceptive receptors [16]. Therefore, capsaicin can effectively be used to induce central sensitization.

Capsaicin will be applied directly to the region of the innervation zone at the muscle belly to sensitize the neurons within the region of taut band development. Topical and intramuscular capsaicin will be used. The capsaicin formula will be compounded by a registered pharmacist. Topical capsaicin will be delivered in a cream and injectable capsaicin will be intermixed with saline prior to injection (2 cc). The control group in the topical capsaicin arm will be treated with the cream base used during the experiment without added capsaicin and the injection arm will be injected with saline. A trained medical professional on the research team will apply the topical capsaicin or topical placebo treatments. The region of topical application will measure 5 × 5 cm square in the dermatome zone location to cover an area of approximately 25 cm^2^. A trained physician in physical medicine and rehabilitation will deliver injectable capsaicin using a 27-gauge needle at the location of the superior fascia of the upper trapezius muscle with ultrasound guidance. The fascial layer was chosen as it is the region with the lowest pressure pain threshold relative to skin and muscle, suggesting a high density of nociceptors within the layer, and it possesses connections to the spinal cord [34]. Changes in muscle fascia properties and nociception at muscle fascia, compared to nociception at muscle or subcutaneous tissue, have also been implicated in the development of pain [35, 36]. Furthermore, intramuscular capsaicin will also be injected using ultrasound guidance to avoid the superior or inferior fascia.

To confirm the presence of central sensitization, brush allodynia will be used to detect mechanical hyperalgesia outside the region of primary nociception—region of topical placement or injection—which is the region of secondary hyperalgesia [16–18]. The size of the region of secondary hyperalgesia will be measured to characterize the extent of central sensitization. This will be accomplished using a tape measure and the perpendicular dimensions of the region of secondary hyperalgesia will be recorded in square centimeters.

### Ultrasound analysis

The Sonosite X-Porte Ultrasound machine located at the Toronto Rehabilitation Institute clinic will be used. A linear wave 15-6 MHz probe will be placed onto the trapezius before and after the sensitization is induced (approximately 20 min) and an ultrasonic image will be captured. Texture feature analysis will be performed using MATLAB and the Signal Processing Toolbox. Analyses will include first order parameters such as mean and standard deviation of the pixel level values, as well as second order parameters, which provide details on the spatial distribution of pixel values. These include co-occurence and run-length matrices, local binary pattern, and blob analysis [37].

#### Measurement of motor unit activity using EMG

The Delsys Trigno surface EMG system will be used in this experiment. The system offers the benefit of having a 4-pin mini-grid that can extract individual motor unit data, without causing participants discomfort. The analysis will also be complemented by the use of intramuscular EMG, since it offers high location specificity when measuring EMG activity, while only a local region is analyzed. The intramuscular EMG data will be analyzed by a Cadwell.

### Sierra wave device

The anatomical location for electrode placement, along the C7-acromion line, will be identified for each participant. Baseline EMG measurements, as well as the ultrasonic images, will be taken from the upper trapezius muscle to obtain the baseline motor unit recruitment curve. The trapezius muscle was chosen for study since it is a common site for MTrPs, given the high load cervical muscles carry as well as the general increase sedentariness that perpetuates poor posture and cervical muscle strain, leading to trigger point presentation. Furthermore, the trapezius muscle has been used frequently in previously published work, which analyze motor unit parameters, power spectrum, and amplitude measurements [2, 38, 39].

#### sEMG analysis

After the identification for electrode placement and the captured baseline ultrasonic images, the area will be cleaned with alcohol and water. The Delsys Galelio sensor will be placed directly on the identified area. The EMG sensor (bandwidth of 20–450 Hz) consists of 4 metal contacts for detecting the signal at the skin surface. Both the right and left sides of the trapezius will be measured.

The Delsys Trigno system, which includes sensors, the Trigno base station, and the EMGworks software, will be used to decompose the acquired EMG signals into individual motor units. The following parameters will be analyzed: the firing times and frequency of individual motor units, motor unit action potential amplitude and shapes [40–42], the root mean square value (RMS) of each channel and of the entire signal [22], the coefficient of variation for force steadiness [43], and the centroid of the EMG signal in the cranial-caudal and the medial-lateral directions [22]. This will be repeated for each contraction.

#### Intramuscular EMG analysis

A 27-G monopolar electromyography needle will be placed into the midbelly of the upper fibers of the trapezius muscle using ultrasound guidance. The intramuscular EMG signals will be measured using Cadwell Sierra Wave. Since this system does not offer motor unit decomposition, we will use the intramuscular EMG analysis to provide us with more details of the gross EMG measurements such as the overall RMS value, the coefficient of variation for force steadiness, and the centroid measurements.

### Determination of central sensitization

Before measuring outcomes, approximately 20 min after capsaicin application, we will analyze the area of secondary hyperalgesia for each study subject and then determine whether central sensitization has in fact been induced. The presence of central sensitization is confirmed by expansion of the receptive field and area of secondary hyperalgesia beyond the 25 cm^2^ initial application area. If it has, the study participant will undergo the rest of the experimental methodology. If it has not, the capsaicin will be applied once more and the region of secondary hyperalgesia will be re-measured 20 min later. The above-described process for confirming the presence of central sensitization will take place again. If the study participant has not had sensitization at this stage, they will be withdrawn from the study.

#### Primary outcomes

The primary outcome measure is as follows:

1. Recruitment and amplitude changes before and after capsaicin application

**Fig. 2.**
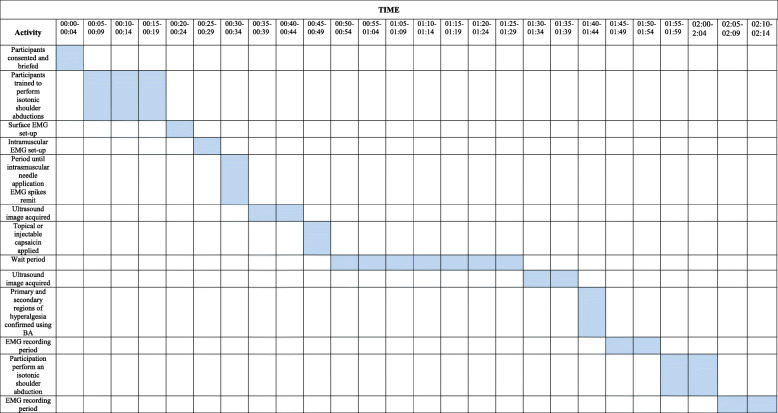
Flow chart of methods

These will be recorded for each arm of the study, please see Fig. 2.

Secondary outcomes are as follows:

1. Capsaicin-induced sensitization affecting the rate of recruitment of motor units comparison before and after of capsaicin application.
2. Ultrasound textural regional changes before and after capsaicin application
3. Capsaicin-induced sensitization recording electrical recording at the MTrP with comparison before and after.
4. A record of adverse events
5. Safety evaluation

These will be recorded for each arm of the study, please see Fig. 2.

The investigating physician monitors the study participant clinically for signs of distress. Any signs of distress noted by the investigating physician will be assessed clinically and appropriately managed. The information will be recorded on the study participant’s data form. The study team will discuss all incidents and any potential causal link to the study interventions. Participants will be provided with a contact number for the principal investigator in order to report any changes that occur after the study. Our publication will include a list of any adverse events or intercurrent illnesses encountered. Any study participant who experiences an adverse event will be triaged to their family physician or local emergency department as necessary. They will be contacted after to obtain information on the course of their treatment and the outcome of the event.

### Sample size calculation

Using GPower V3.0.10 (Dusseldorf, Germany), considering a medium effect size (Hedge’s G) of 0.5 and a moderate correlation coefficients (0.5) among repeated measures, for electrodiagnostic recordings, sample size calculation determined that a minimum of 78 participants would be required to detect differences via repeated measures ANOVA (two measurements as before and after among 9 groups) with 80% power and an alpha of 0.05. We aim to recruit 98 participants to account for potential attrition (~ 20%) of participants given the study duration. We plan to recruit equal number of men and women. To date, there are no reported sex differences in induction of central sensitization using capsaicin among males and females with neurological impairments.

### Statistical analysis plan

Baseline participants’ characteristics including demographics will be analyzed using appropriate descriptive statistics. Mean and standard deviation will be calculated for continuous variables. Categorical variables will be presented as numbers and percentages. This is exploratory research and we are unsure of the characteristic of the dataset that will be obtained; we plan to focus on estimating effect sizes and confidence intervals with the assistance of a biostatistician. The biostatistician will create statistical models to assist in answering our research questions. We plan to evaluate the difference between the forms of delivery (topical, intrafascial, intramuscular) using a 2-way model with consultation by a biostatistician.

All statistical analyses will be conducted with SAS for Windows (version 9.3; SAS Institute, Inc., Cary, NC). Using a two-sided test, a *P* value ≤ 0.05 will be considered as statistically significant.

## Discussion

This will be the first study that assesses the effect of capsaicin-induced central sensitization on ultrasound texture features and muscle motor unit activity. Findings from this study may support one of few hypotheses proposed delineating the involvement of central sensitization in the development of trigger points. A prominent hypothesis in the literature is the integrated hypothesis. The integrated hypothesis suggests that unaccustomed eccentric activity or submaximal to maximal concentric muscle exertion leads to muscle fiber damage, segmental hypercontraction within the muscle fibers, and ischemia [9]. The resultant damage and ischemia at the muscle instigate an inflammatory biomarker cascade that potentiates the activity of motor neurons, subsequently increasing the release of acetylcholine and inducing muscle contraction, and sensitizes sensory neurons [9, 44]. The sensitization of peripheral sensory and motor neurons is thought to contribute to the sensitization of dorsal horn neurons in the associated spinal segment, which likely influences efferent neuronal activity at the muscle. From this hypothesis, it would be expected to observe uncoordinated motor unit activity and activation given that muscle fiber damage has occurred and motor units may be differentially potentiated or sensitized. The findings from studies inducing acute nociception in the muscle support this hypothesis [22, 45].

Hypertonic saline injections have been shown to reorganize muscle activity within the muscle [45]. Previous findings also suggest that motor unit discharge frequency changes variably among motor units, although predominantly decreasing, as a consequence of external nociception [22]. This study should elucidate whether a nociceptive stimulus that causes central sensitization induces uncoordinated motor unit activation and differential discharge patterns among the units. The integrated hypothesis presupposes that the cause of myofascial trigger points is exogenous and induces endogenous changes in muscle. The Cinderella and the neurogenic hypothesis suggest otherwise.

The Cinderella hypothesis suggests sustained low threshold motor unit activation metabolically overloads oxidative muscle fibers, leading to muscle fiber damage or “ragged fiber” presentation and metabolic changes such as ischemia, hypoxia, and insufficient adenosine triphosphate (ATP) [10, 44]. This may result in disturbed calcium homeostasis and the development of the muscle contracture known as the trigger point. Low threshold motor unit activity was demonstrated in a number of articles assessing the effect of continued muscle contraction and psychologically demanding tasks—muscle overexertion and persistent psychological stress are theorized triggers for chronic pain and trigger point development [46, 47]. The sustained contracture in the muscle maintains the hypoxic and acidic environment, leading to peripheral and central sensitization of neurons in the associated spinal segment [10]. This suggests an endogenous and exogenous cause for the trigger points. Under the premise of the Cinderella hypothesis, it would be expected to observe sustained low threshold motor unit activity with central sensitization.

The neurogenic hypothesis is distinct from the integrated and Cinderella hypotheses as it poses trigger point development as a result of endogenous central sensitization [1]. Central sensitization would result from persistent nociceptive input from other peripheral mechanic or systemic pathologies leading to neurogenic inflammation, such as trauma or endocrine disease.

Subsequently, inflammatory and algogenic substances would be released from peripheral nociceptors onto a tissue and sensitizing the region. Sensitization of motor and sensory neurons innervating somatic muscle may lead to the phenomenon of trigger points. Ultimately, the neurogenic hypothesis stipulates the trigger point is a secondary outcome central sensitization, which is the primary pathology. Continuous low threshold motor unit activity would also be an anticipated observation as neurons will be increasingly sensitized. It would also be expected to observe increased low threshold motor unit activity in remote muscles that present with signs of central sensitization, e.g., hyperalgesia; however, this is outside the scope of this article.

## Limitations and conclusions

Overall, the findings from this study should present preliminary evidence to inform central sensitization’s effects on motor unit activity. Results may provide plausibility for the aforementioned hypotheses. A limitation of this study is the degree of invasiveness needed to increase the specificity of the EMG measurements; however, the trade-off between invasiveness and specificity is necessary to ensure we capture a sufficient effect size with a feasible sample size. Additionally, the experimental protocol will be carried out on healthy participants with induced central sensitization to determine the effects of this phenomenon on muscle activity.

Although these results are not directly generalizable to participants with myofascial pain, this study’s results should provide insight as to whether central sensitization does perturb muscle activity and structure, and inform further investigations into the pathophysiology of myofascial pain. Another limitation of this study in light of the integrated and Cinderella hypotheses is that sensitization will be induced prior to the assessment of EMG activity at motor neurons.

However, it is important to understand whether central sensitization is a direct cause of aberrant motor unit activity, as a fraction of the population develops myofascial trigger points following precipitating events such as injury, stress, or poor posture. The presence of central sensitization at a specific severity or “tipping point” may predispose some individuals to developing trigger points—or induce trigger points with increased severity—and the mechanisms described by the integrated or Cinderella hypotheses may lead to the manifestation of the trigger point. These hypotheses need not be mutually exclusive, but rather may be complementary or present as alternate etiologies to the development of the trigger point. It is possible the anatomical changes resulting from each hypothesis lead to a different manifestation of trigger points that appear clinically equal. Further studies assessing central sensitization markers and EMG activity of muscle innervated by spinal segments remote from the region of primary sensitization are needed to elucidate the overarching pathophysiology of trigger point presentation.

## Supplementary Information

**Additional file 1.**

## Data Availability

The datasets used and/or analyzed during the study will be available from the corresponding author upon reasonable request.

## Abbreviations

ATP: Adenosine triphosphate
EMG: Electromyography
IASP: International Association for the Study of Pain
MPS: Myofascial pain syndrome
MTrP: Myofascial trigger points
MUAP: Motor unit action potential
RMS: Root mean square
SEA: Spontaneous endplate activity
UHN: University Health Network
US: Ultrasound
