# Supplementary material for: Quantitative response of healthy muscle following the induction of capsaicin: an exploratory randomized controlled trial
Source: Trials. 2020 Dec 11;21:1020. doi: 10.1186/s13063-020-04937-4 (PMC7731533; doi:10.1186/s13063-020-04937-4)
Supplement: Supplementary file 1 — Additional file 1. [file 13063_2020_4937_MOESM1_ESM.pdf]

University Health Network
Research Ethics Board
10th Floor, Room 1056
700 University Ave.
Toronto, Ontario, M5G 1Z5
Phone: (416) 581-7849

NOTIFICATION OF REB INITIAL APPROVAL

Date: February 26, 2019

To: Dinesh Kumbhare
Toronto Rehabilitation Institute, University Centre, 550
University Avenue, Suite 7-131, Toronto, Ontario,
Canada, M5G 2A2

Re: 18-6163
Assessing Electromyographic Responses of Healthy
Muscle Following the Induction of Central
Sensitization using Capsaicin: A Randomized
Controlled Trial

REB Review Type: Full Board
REB Meeting Date(s): December 10, 2018
REB Initial Approval Date: February 26, 2019
REB Expiry Date: February 26, 2020

Documents Approved:

Document Name	Version Date	Version ID
Consent Form	February 8, 2019	3
Data Collection Sheet	November 5, 2018	1
Email Script	February 14, 2019	2
Protocol	January 2, 2019	2
Flyer	January 2, 2019	2

The University Health Network Research Ethics Board approves the above mentioned study as it has been found to comply with relevant research ethics guidelines, as well as the Ontario Personal Health Information Protection Act (PHIPA), 2004.

Best wishes on the successful completion of your project.

Sincerely,
Morris Sherman
Co-Chair, University Health Network Research Ethics Board

The UHN Research Ethics Board operates in compliance with the Tri-Council Policy Statement; ICH Guideline for Good Clinical Practice E6(R1); Ontario Personal Health Information Protection Act (2004); Part C Division 5 of the Food and Drug Regulations; Part 4 of the Natural Health Products Regulations and the Medical Devices Regulations of Health Canada. The approval and the views of the REB have been documented in writing. The REB has reviewed and approved the clinical trial protocol and informed consent form for the trial which is to be conducted by the qualified investigator named in the letter.
Furthermore, members of the Research Ethics Board who are named as Investigators in research studies do not participate in discussions related to, nor vote on such studies when they are presented to the REB.
